# The Role of Enjoyment in a Serious Game for Binge Drinking Prevention: Pretest-Posttest Quasi-Experimental Study

**DOI:** 10.2196/21652

**Published:** 2020-11-30

**Authors:** Traci Hong, Joshua Cabrera, Christopher E Beaudoin

**Affiliations:** 1 College of Communication Boston University Boston, MA United States; 2 MHMR Authority of Brazos Valley Bryan, TX United States

**Keywords:** alcohol prevention, binge drinking, serious game

## Abstract

**Background:**

Although binge drinking peaks at age 21 to 25 years, there is limited research on the effects of serious games in this population, as well as on the process by which playing serious games impacts alcohol-related outcomes. Designed with both health behavioral theory and game theory, One Shot is an online serious game that aims to prevent binge drinking.

**Objective:**

This study utilized a conceptual model for serious video game processes. Using One Shot, the model assessed the following process stages: (1) Alcohol Use Disorders Identification Test-Concise (AUDIT-C); (2) in-game factors of game time and risky alcohol decisions; (3) game enjoyment; and (4) postgame outcomes of intention to drink less and drinking refusal self-efficacy.

**Methods:**

In a one-group pretest-posttest quasi-experimental design, a sample (N=550) of young adults (age 21-25 years) who reported recent binge drinking played the One Shot game. Intention to drink less and drinking refusal self-efficacy were measured at pregame and postgame, with their effects lagged in statistical analysis. Participants were presented with various scenarios in the game that pertained to risky alcohol decisions, which, along with game time, were unobtrusively recorded by the server. A structural equation model (SEM) was used to test the conceptual model, with assessments made to determine if enjoyment mediated the effects of game time and risky alcohol decisions on the 2 postgame alcohol-related outcomes.

**Results:**

A well-fitting SEM demonstrated support for the multistep model, with AUDIT-C predicting risky alcohol decisions (β=.30). Risky alcohol decisions (β=−.22) and game time (β=.18) predicted enjoyment, which, in turn, predicted intention to drink less (β=.21) and drinking refusal self-efficacy (β=.16). Enjoyment significantly (*P*<.001) mediated the effects of game time and risky alcohol decision on intention to drink less and drinking refusal self-efficacy.

**Conclusions:**

The results support a conceptual model in which staggered individual and in-game factors influence alcohol-related outcomes. Enjoyment is important for participants’ intentions to drink less and beliefs that they can refuse alcohol.

## Introduction

### Background

Although it is well recognized that alcohol use is a leading risk factor for morbidity and mortality, recent estimates indicate that it accounts for a larger percentage of global deaths than previously recognized, with 10% of deaths among 15 to 49-year-old individuals attributed to alcohol [1]. Excessive alcohol use is associated with considerable health burdens and outcomes, including cancer, heart disease, and stroke [2-4]. Binge drinking, which is defined as consuming 5 or more drinks for men and 4 or more drinks for women in a 2-hour period [5], is common, with more than a quarter of US adults reporting it [6]. To prevent alcohol misuse, video games have been used to impart knowledge and change health behaviors [7], with much of this research focused on adolescents and college students because of the health and injury risks associated with underage drinking [8]. Designed to entertain, as well as simultaneously impart knowledge and model health behaviors [9,10], these “serious video games” are interactive interfaces with a single player or multiple players on different platforms, including online, computer, game console, and mobile [10].

While there are several serious games on substance use, few are exclusively focused on alcohol, with nearly all such games targeting adolescents and college students [8,11-13]. Research on serious games has demonstrated participants' development of knowledge on harms and attitudes about alcohol consumption [8]. In terms of behavioral change, there is evidence that serious games can result in increased drinking refusal skills [8] and short-term effects on reduced alcohol consumption [13], particularly among females [12] and younger adolescents [12]. In contrast, some studies found inconclusive effects [14] or no effect [11].

There are very few studies specific to adults of legal drinking age (ie, 21 years or older) in the United States. Though understudied, young adults (ie, from 21 to 25 years of age) represent an important population for alcohol-prevention gaming because excessive alcohol consumption peaks at the end of or even after college, with binge drinking levels at a high for women at age 22 years and for men at age 23 years [15]. These peak ages for binge drinking reflect a steady increase across the last 30 years, whereby the peak age of binge drinking has increased by 2 years [15]. In addition, the highest weekly alcohol intake occurs at about age 25 years for both men and women [16]. Young adults are also an important group for alcohol-prevention video games given that the majority of people in this age group have played video games [17].

The aim of this study was to explain how individual characteristics and in-game factors of a serious game can influence drinking-related outcomes for young adults aged 21 to 25 years. To study the underlying mechanisms, we build a multistep model that entails individual factors, in-game factors, and postgame alcohol outcomes. Below, we describe our serious video game and its theoretical basis. Then, we articulate expectations related to our conceptual model.

### “One Shot” Online Video Game

**Figure 1:**
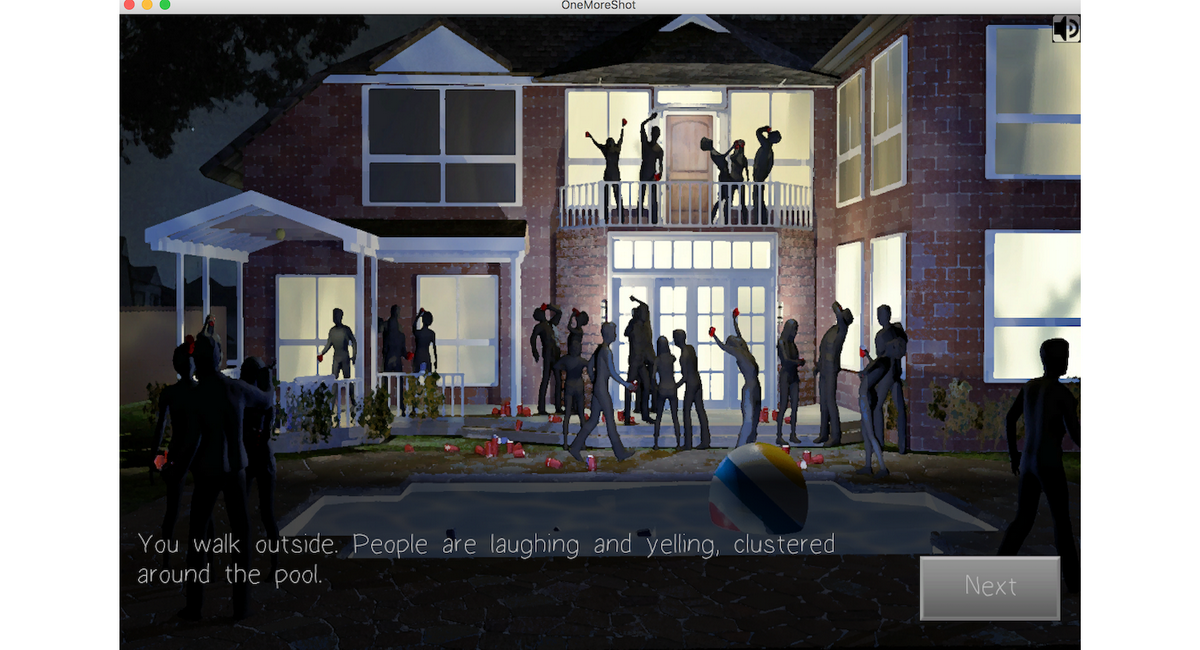
Screenshot of the party scene of the One Shot game.

This study’s serious game “One Shot” is an online single-player game designed by a physician (ie, this study’s second author) specializing in alcohol addiction among young adults. Single-player games can be particularly adept at imparting new skills and knowledge [18]. One Shot is a 2-dimensional web-based game created on the Unity Game platform with a role-playing story-based design in which players make decisions that influence their relationships with other game characters. The game characters are nonplayer characters who the game player does not control. Subtle background noise (eg, chatter of people at a party) and music appear throughout the game. The dialogue and narrative description are displayed in a box at the bottom of the screen. Player interaction is facilitated with mouse clicks or keyed selections. The player advances through the narrative description and makes decisions by selecting the preferred option. The dialogue was pilot tested with 21-year-old individuals. The game can be run on the Chrome or Firefox browser. Figure 1 depicts the scene game players encounter upon arriving at a party.

#### Theoretical Basis

**Figure 2:**
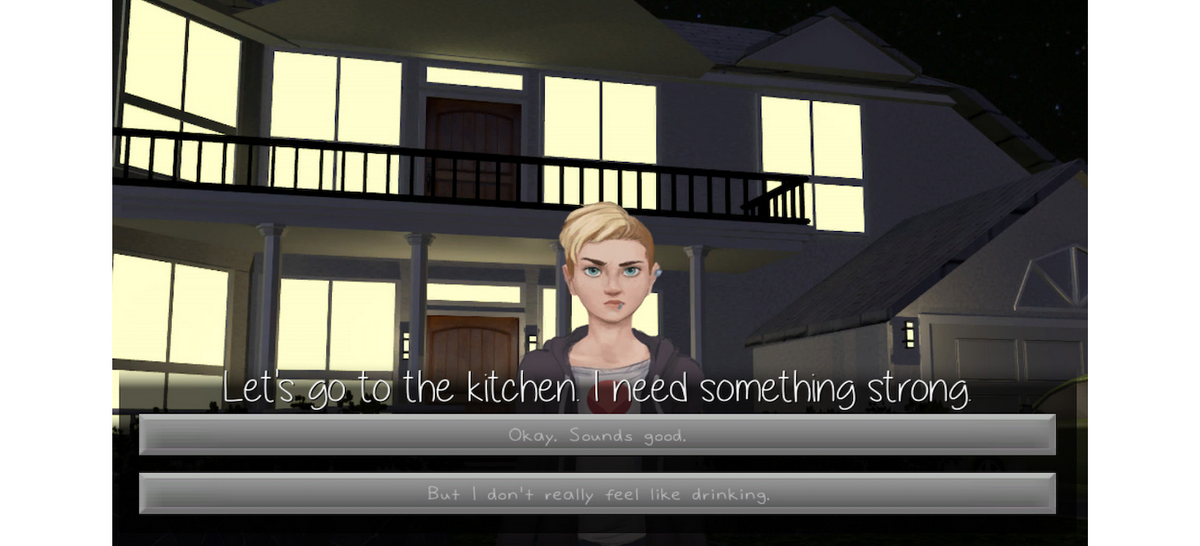
Screenshot of a close friend nonplayer character encouraging the game player to drink strong alcohol.

The design of One Shot utilizes both game theory and health behavioral theory given that the combination of both theoretical approaches elicits the strongest health outcomes [19]. In terms of game theory, central to an effective serious game is a story-telling element [9,10]. In particular, role-playing games are adept at facilitating a video game player’s identification with characters and roles [20]. This type of identification can subsequently lead to game enjoyment [20], which is important to alcohol-related outcomes. The video game narrative begins when two close friend nonplayer characters arrive to pick up the game player to go to an evening party at a house where alcohol is widely consumed by guests. Conflict emerges when the game player is exposed to the opposing perspectives on alcohol consumption of the two friends and needs to choose between varying levels of risky alcohol consumption. Figure 2 depicts one of the close friend nonplayer characters at the party and an example of a high-risk alcohol consumption opportunity (ie, consumption of strong alcohol) that is presented to game players. Irrespective of the risk decisions made in One Shot, all game participants experience a car accident involving a physical injury and visit to the emergency room, thus highlighting the severe negative consequences of drinking. The game player and close friend nonplayer characters meet a nonplayer character nurse in the emergency room, who explains that a car accident occurred and then educates them about the hazards of binge drinking.

**Figure 3:**
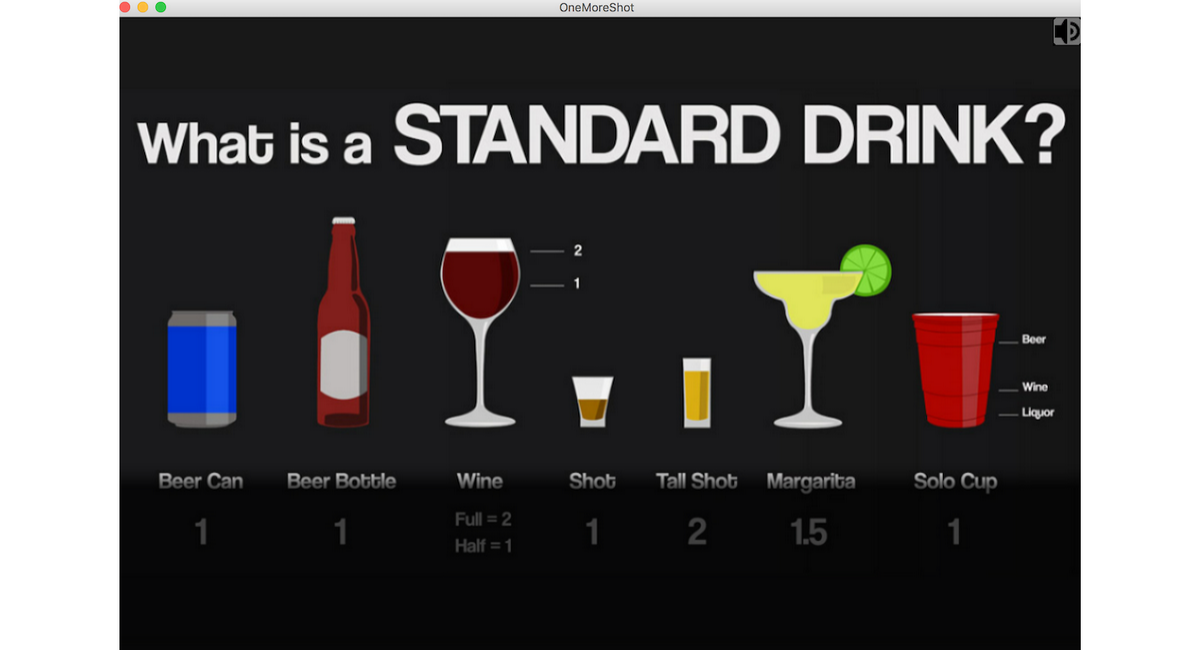
Screenshot of tailored feedback for game players.

In terms of health behavioral theory, protection motivation theory (PMT) has the following 2 core concepts: perceived threat and coping. It suggests people are likely to take action to avert a severe threat if they are provided with coping strategies [21]. In the emergency room scene of One Shot, coping strategies are conveyed by tailored feedback that is given to game players after they are asked about their alcohol-related knowledge and perceptions. For example, Figure 3 depicts tailored feedback provided to game players who do not understand the size of standard alcoholic drinks. Other instances of tailored feedback entail specific coping strategies to avert binge drinking.

In addition, given that previous research has demonstrated that social norms influence alcohol consumption [22,23], One Shot aims to utilize the normative influence of close friend nonplayer characters. The game provides a mix of peer approval and disapproval of alcohol consumption. In particular, there is strong disapproval from the nurse, whereas, of the two friends at the beginning of the game, one strongly approves of binge drinking and the other disapproves of binge drinking. Across the game, the nonplayer character friend who was initially prodrinking changes to disapprove of binge drinking. With this transition, both nonplayer character friends, as well as the nonplayer character nurse, strongly disapprove of binge drinking by the end of the game.

### A Conceptual Model for Serious Video Game Processes

**Figure 4:**
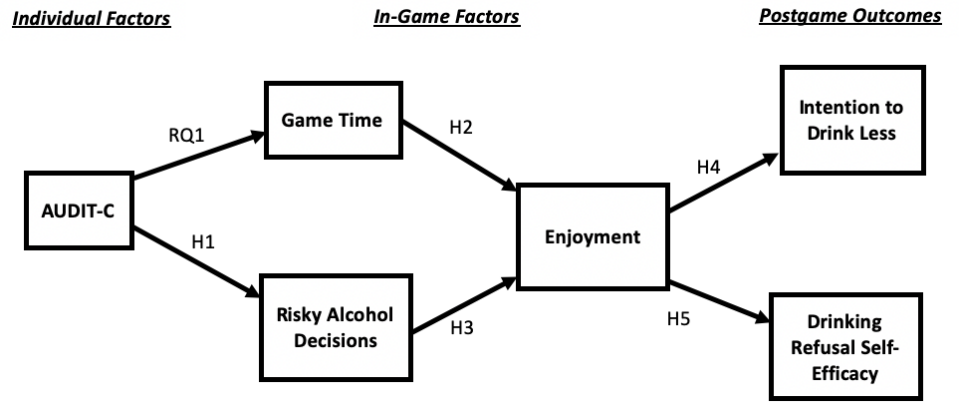
Conceptual model for serious video game processes.

Our conceptual model for serious video game processes is depicted in Figure 4. It entails the following 3 main stages of factors: (1) individual factors; (2) in-game factors; and (3) postgame outcomes. In-game factors have 2 substages entailing time spent playing the game and risky alcohol decisions that are made (both of which are unobtrusively recorded by the host server) and enjoyment of the game (which is measured immediately after the game).

#### Individual Factors

The first set of factors in our conceptual model is the individual characteristics of the game player. Prior research has implemented health predispositions as an individual factor [24]. For example, AUDIT-C was implemented as an individual factor for a serious game that assessed alcohol outcomes and participants’ willingness to seek help for health problems [13]. AUDIT-C measures risk for hazardous alcohol consumption and alcohol use disorder [25]. These studies suggest that measures of health disorders, such as AUDIT-C, are individual factors that can affect how people play serious video games and are influenced by them.

#### In-Game Factors and Their Antecedents

The second main stage in our conceptual model entails in-game factors specific to the One Shot serious game. In Figure 4, they appear in the following 2 substages: (1) game time and risky alcohol decisions and (2) enjoyment. In the first substage, game time entails how long a player was active in the game environment, while risky alcohol decisions involve how many risky choices game players made when presented with various scenarios that entailed alcohol consumption, including low-risk decisions such as drinking one alcoholic beverage, but refusing another, and high-risk decisions such as elevating drinking to hard liquor, playing a drinking game, and drinking and driving.

Pertinent to this stage in the model, research indicates that video game players behave in a virtual environment similarly to how they behave in the real world [26]. Thus, we expect that individuals who exhibit hazardous drinking in real life will exhibit similar drinking behaviors in the game. Given that AUDIT-C is a reliable measure for alcohol abuse disorder and hazardous drinking [27], which is associated with risky decision making [28], we expect that game players who are more likely to exhibit hazardous drinking (ie, higher levels of AUDIT-C) will choose to partake in more risky alcohol behaviors when they are presented with such options in the video game. The first hypothesis (H1) is as follows: AUDIT-C predicts an increase in risky alcohol decisions.

Unclear, however, is the effect of hazardous drinking on time spent playing the game. The first research question (RQ1) is as follows: What is the effect of AUDIT-C on game time?

The second substage of in-game factors (Figure 4) entails enjoyment of the game. Important to media entertainment on any platform (eg, video game, film, and television) is a user’s sense of enjoyment, which is defined as a positive reaction to media content [29], encompassing both cognitive and affective dimensions [29]. A fundamental aspect of serious games is that learning and health behavior change occur alongside entertainment [9,10], that is, video game players are entertained while, through social modeling [30], they acquire knowledge, which can lead to behavioral adoption.

Despite the suggested importance of enjoyment, few serious games specific to alcohol prevention account for the role of enjoyment in influencing related game outcomes. In our conceptual model, the in-game factors of game time and risky alcohol decisions predict game enjoyment. Previous research found that in-game experiences are associated with enjoyment [31]. Additionally, previous research documented that the more time people spend playing a video game, the more likely they are to report enjoying the game [32]. The second hypothesis (H2) is as follows: Game time predicts an increase in enjoyment.

Next, we considered the effects of risky alcohol decisions. In the One Shot game, players who make more risky alcohol decisions are more extensively exposed to game scenarios that lead to the game’s severe consequences (eg, car accident and visit to a hospital). These severe consequences serve as fear appeals, which elicit an unpleasant emotional state that arises from a threat [33], and in PMT, result from perceptions of the severity and susceptibility of the threat [21]. In the video game, the severity of the threat encompasses the car accident and emergency room visit, whereas the susceptibility of the threat entails the escalating storylines as players make more risky alcohol decisions. Making more risky decisions would thus likely elicit negative emotions and, in turn, lower levels of game enjoyment. The third hypothesis (H3) is as follows: Risky alcohol decisions predict a decrease in enjoyment.

#### Postgame Outcomes and Their Antecedents

The third stage in our conceptual model (Figure 4) entails the following 2 postgame outcomes: intention to drink less and drinking refusal self-efficacy. Behavioral intention is defined as how likely a person thinks he/she would perform a specific behavior and is highly predictive of a person actually performing such a behavior in the future [34]. Drinking refusal self-efficacy is conceptualized as one’s belief in his/her ability to refuse solicitation of alcohol in commonly encountered situations (eg, when a friend dares you to drink in excess) [35] and is highly predictive of alcohol consumption frequency and volume [36].

We consider the effects of enjoyment on both of these outcomes. Enjoyment of a game underpins the entertainment media experience, such that attaining game enjoyment is necessary for substantive effects, most notably learning outcomes [29]. Previous research on serious games has documented the importance of game enjoyment, with people’s enjoyment of a game being correlated with their positive attitudes toward the game [37], as well as their self-efficacy [38] and intention to perform a recommended behavior [39]. Given that we lag the postgame outcomes by analogous measures at pregame, our hypotheses pertain to the change in outcomes. The fourth hypothesis (H4) is as follows: Enjoyment predicts postgame intention to drink less (when controlling for its pregame level). The fifth hypothesis (H5) is as follows: Enjoyment predicts postgame drinking refusal self-efficacy (when controlling for its pregame level).

Given the multistage aspect of our conceptual model for serious video game processes (Figure 4), we also considered the mediation role played by enjoyment. Because enjoyment is a central concept in entertainment media [29] and entertainment serves as the foundation for learning in serious games [9,10], it is expected that enjoyment plays an integral role in the One Shot game, mediating the effects of prior in-game factors on postgame outcomes. The sixth hypothesis (H6) is as follows: Enjoyment mediates the effects of game time on postgame intention to drink less and drinking refusal self-efficacy (when controlling for the pregame levels). The seventh hypothesis (H7) is as follows: Enjoyment mediates the effects of risky alcohol decisions on postgame intention to drink less and drinking refusal self-efficacy (when controlling for their pregame levels).

## Methods

### Sample and Design

This study utilized a one-group pretest-posttest quasi-experimental design [40], which has been used to evaluate serious video games for alcohol prevention [13], as well as other health education topics [41,42]. The One Shot video game was implemented online and made accessible to study participants. There were online survey assessments before (ie, pregame) and after (ie, postgame) the online video game. Measures for 2 in-game factors (ie, game time and risky alcohol decisions) were captured through log files on the host server. The sample of participants was recruited by Survey Sampling International (SSI) and is of US young adults aged 21 to 25 years who reported binge drinking in the 2 weeks preceding this study (N=550). SSI drew participants from a nationally representative internet panel of individuals from all 50 states, and the sample is representative of national demographic parameters. To build the panel, the SSI panel used targeted approaches, including search links, banner advertisements, email, online invitations, and coregistration. SSI also employed data-validation techniques, such as comparing participant demographics with multiple databases and data vendors, to verify personal identifying information. The pregame and postgame questionnaires were conducted via Qualtrics in June and July 2016. Institutional Review Board approval for this study was acquired at Texas A&M University.

### Measurements

There were the following 4 demographics: age, education, ethnicity (W=1), and gender (M=1). Education was measured on a 7-point scale from less than high school graduate (1) to doctoral degree (7) and recoded to represent years of study. Other variables were AUDIT-C, game time, risky alcohol decisions, enjoyment, intention to drink less, and drinking refusal self-efficacy. AUDIT-C was assessed at pregame, whereas enjoyment was assessed at postgame. Intention to drink less and drinking refusal self-efficacy were assessed at pregame and postgame.

With scores ranging from 0 to 12, AUDIT-C entails 3 survey items that help identify people who are hazardous drinkers or have active alcohol-use disorders [25]. Game time was measured in the following 4 ordered groups: (1) 0-500 seconds; (2) 501-1000 seconds; (3) 1001-1500 seconds; and (4) 1500-1800 seconds. Risky alcohol decisions involved the following 2 items: low-risk alcohol decisions and high-risk alcohol decisions. There were 2 survey items for enjoyment [43]. The first question entailed how much participants enjoyed the game experience, with responses on a 5-point scale from “I really didn’t enjoy it” (1) to “I really enjoyed it” (5). The second question entailed the likelihood of participants recommending the game to a friend, with responses on a 5-point scale from “strongly recommend” (1) to “strongly not recommend” (5). This second set of responses was reversed coded, so that higher responses to both questions represented more positive impressions of the video game (*r*=0.54, *P*<.001). Intention to drink less was measured with one item on a 5-point scale (with responses from 0-4), involving whether participants planned to moderate their alcohol use in the future [44]. Drinking refusal self-efficacy was assessed with 7 items [45]. For example, participants were asked how sure they were that they could stop drinking if there were problems with friends or for themselves, with responses on a 5-point scale from “not at all sure” (1) to “extremely sure” (5). This scale was internally consistent at pregame (α=.83) and postgame (α=.88).

### Statistical Analysis

Stata 16 (Stata Corp) was used for statistical analysis. No outliers were identified. Square root transformations were conducted for skewed-right variables (ie, AUDIT-C, game time, and the 2 manifest indicators of risky alcohol decisions). Square transformations were implemented for skewed-left variables (ie, intention to drink less and the 2 manifest indicators of enjoyment). Descriptive statistics, as well as *t* tests comparing pregame and postgame means of the dependent variables, were used for variables in their pretransformation form. Posttransformation variables were used for the structural equation model (SEM), which was implemented using the maximum likelihood method of estimation and covariance structure analysis. SEM is a technique for confirming a specified model of multivariate relationships [46]. Entailing simultaneous regressions, SEM permits the testing of the stage-like effects of our conceptual model for serious video game processes. The excellence of model fit is assessed according to the benchmarks of Hu and Bentler [47] as follows: a comparative fit index (CFI) value ≥0.95 and a root mean square error of approximation (RMSEA) value of close to 0.06 or less.

Exogeneous control variables included age, education, ethnicity (W=1), and gender (M=1), as well as the pregame measures of intention to drink less and drinking refusal self-efficacy. Endogenous variables included AUDIT-C, game time, risky alcohol decisions, and enjoyment, as well as postgame measures of intention to drink less and drinking refusal self-efficacy. Paths were drawn from exogenous variables to endogenous variables and among endogenous variables, as depicted in Figure 4. Using an autoregression approach, paths from pregame to postgame intention to drink less and from pregame to postgame drinking refusal self-efficacy entail cross-lagged effects, which represent the stability of a measure over time and control the postgame variable for the pregame level of the variable [48]. In this manner, the effects of the other exogenous and endogenous variables on postgame intention to drink less and postgame drinking refusal self-efficacy are indicative of their influence on the changes from pregame to postgame in these health outcomes. There were the following 2 covariance paths: between game time and risky alcohol decisions and between postgame intention to drink less and postgame drinking refusal self-efficacy.

In the SEM, enjoyment, risky alcohol decisions, and pregame and postgame drinking refusal self-efficacy were instituted as latent variables, while the other variables were observed. In the first step of the SEM approach, confirmatory factor analysis (CFA) was conducted to test the unidimensionality of the latent variables on pregame and postgame drinking refusal self-efficacy. Second, a structural model was tested to assess relationships among the latent and observed variables. Mediation was assessed with the product of coefficients approach [49].

## Results

Descriptive statistics are depicted in Table 1. In their pretransformation form, the mean for intention to drink less was significantly higher at postgame than at pregame (1.61 vs 1.46; t_549_=−2.97, *P*=.003). Similar tests were run on the 7 pregame and postgame manifest indicators of drinking refusal self-efficacy, which was instituted as a latent variable in the SEM. In the following 5 cases, the item means were higher at postgame than at pregame (Table 1): (1) item 1, t_549_=−5.51, *P*<.001; (2) item 3, t_549_=−5.29, *P*<.001; (3) item 4, t_549_=−5.89, *P*<.001; (4) item 5, t_549_=−8.11, *P*<.001; and (5) item 6, t_549_=−2.65, *P*=.008. In the other 2 cases, the item means did not vary significantly from pregame to postgame (Table 1) as follows: item 2, t_549_=−1.93, *P*=.054 and item 7, t_549_=−0.57, *P*=.57.

**Table 1:** Descriptive statistics (N=550).

Variable		Value
Age (years), mean (SD)		22.65 (1.18)
Education (years), mean (SD)		14.41 (2.10)
AUDIT-C^a^ (0-12 index), mean (SD)		4.11 (2.17)
Game time (1-4 scale), mean (SD)		1.81 (2.17)
Low-risk decisions (number), mean (SD)		1.61 (1.38)
High-risk decisions (number), mean (SD)		3.56 (3.30)
Game enjoyment (1-5 scale), mean (SD)		3.87 (1.13)
Game recommendation (1-5 scale), mean (SD)		4.13 (1.01)
Pregame intention to drink less (0-4 scale), mean (SD)		1.46 (1.36)
Postgame intention to drink less (0-4 scale), mean (SD)		1.61 (1.36)
**Pregame drinking refusal self-efficacy, mean (SD)**		
	Item 1 (1-5 scale)	3.58 (1.26)
	Item 2 (1-5 scale)	4.09 (1.21)
	Item 3 (1-5 scale)	3.55 (1.27)
	Item 4 (1-5 scale)	3.30 (1.36)
	Item 5 (1-5 scale)	3.01 (1.38)
	Item 6 (1-5 scale)	3.89 (1.30)
	Item 7 (1-5 scale)	4.05 (1.22)
**Postgame drinking refusal self-efficacy, mean (SD)**		
	Item 1 (1-5 scale)	3.86 (1.29)
	Item 2 (1-5 scale)	4.18 (1.18)
	Item 3 (1-5 scale)	3.81 (1.23)
	Item 4 (1-5 scale)	3.63 (1.33)
	Item 5 (1-5 scale)	3.49 (1.37)
	Item 6 (1-5 scale)	4.02 (1.21)
	Item 7 (1-5 scale)	4.07 (1.22)
**Ethnicity, n (%)**		
	Other	229 (41.64)
	White	321 (58.36)
**Gender, n (%)**		
	Female	317 (57.64)
	Male	233 (42.36)

^a^AUDIT-C: Alcohol Use Disorders Identification Test-Concise.

CFA included the latent structures for pregame and postgame drinking refusal self-efficacy. The initial CFA included covariance paths between pregame and postgame drinking refusal self-efficacy and between each pregame manifest indicator and its analogous postgame manifest indicator. The tested CFA had decent model fit (CFI=0.944; RMSEA=0.080; χ^2^_69_=312.61, *P*<.001). According to modification indices, 2 additional covariance paths were added between latent variable indicators. The retested CFA had good model fit (CFI=0.977; RMSEA=0.052; χ^2^_67_=167.65, *P*<.001). This latent variable structure, with factor loadings on the 2 factors, was used in the structural model, which was calculated next. The tested SEM had good model fit (CFI=0.960; RMSEA=0.039; χ^2^_246_=449.08, *P<*.001). The Bentler-Raykov squared multiple correlation coefficients were as follows: AUDIT-C, 5.58%; game time, 2.01%; risky alcohol decisions, 11.53%; enjoyment, 17.22%; postgame intention to drink less, 40.57%; and postgame drinking refusal self-efficacy, 70.35%.

**Figure 5:**
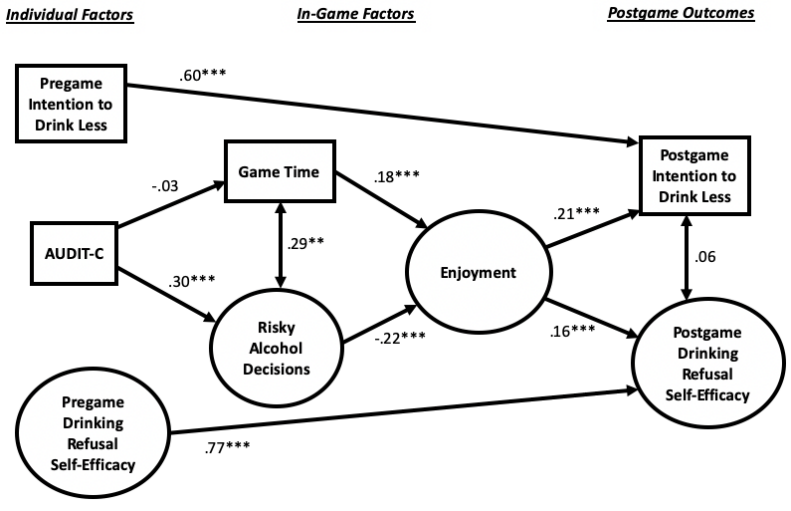
Direct effects of variables in the structural equation model (N=550). *P<.05, **P<.01, ***P<.001.

The effects of variables are depicted in Figure 5. Both lagged effects were significant (intention to drink less, β=.60, *P*<.001; drinking refusal self-efficacy, β=.77, *P*<.001), which suggests over-time stability in measurement. The positive effect of AUDIT-C on risky alcohol decisions was significant (β=.30, *P*<.001), supporting H1. In terms of RQ1, the effect of AUDIT-C on game time was not significant (β=−.03). The positive effect of game time on enjoyment was significant (β=.18, *P*<.001), supporting H2. The negative effect of risky alcohol decisions on game enjoyment was significant (β=−.22, *P*<.001), supporting H3. The positive effect of enjoyment on postgame intention to drink less was significant (β=.21, *P*<.001), supporting H4. The positive effect of game enjoyment on postgame drinking refusal self-efficacy was significant (β=.16, *P*<.001), supporting H5.

H6 and H7 entail mediation paths. The significant role of enjoyment in mediating the effect of game time on intention to drink less was documented, with a product of coefficients of 13.28 (*P*<.001) [50]. Similarly, the significant role of enjoyment in mediating the effect of game time on drinking refusal self-efficacy was demonstrated, with a product of coefficients of 11.98 (*P*<.001) [50]. These findings support H6. The significant role of enjoyment in mediating the effect of risky alcohol decisions on intention to drink less was demonstrated, with a product of coefficients of −7.78 (*P*<.001) [50]. Similarly, the significant role of enjoyment in mediating the effect of risky alcohol decisions on drinking refusal self-efficacy was documented, with a product of coefficients of 7.02 (*P*<.001) [50]. These findings support H7.

## Discussion

### Principal Findings

Our conceptual process model depicts how a serious game for alcohol prevention influences changes in alcohol-related outcomes. With our sample of young adults aged 21 to 25 years, which is an understudied yet at-risk group for binge drinking, the analysis suggested improvements from pregame to postgame in intention to drink less, as well as in 5 of the 7 manifest indicators of drinking refusal self-efficacy. The SEM approach supports the multistep conceptual model, moving from individual factors to 2 staggered substages of in-game factors to postgame alcohol outcomes. In a practical sense, the effects on intention to drink less and drinking refusal self-efficacy are important given their likelihood of predicting subsequent decreases in alcohol consumption behavior [34,36]. Of theoretical importance, our process model contains conceptual components from both health behavioral and game theories given that research has demonstrated that this joint utilization enhances the likelihood that serious games achieve their desired outcomes [19]. Importantly, the results pertain to an at-risk population as all study participants reported binge drinking during the prior 2 weeks.

Consistent with game theory, the extent that game players enjoyed One Shot elicited increases from pregame to postgame in their intention to drink less and drinking refusal self-efficacy. Notably, enjoyment mediated the effects of the following 2 in-game factors: game time and risky alcohol decisions. Thus, instead of these in-game factors having direct effects on alcohol outcomes, their documented influence was indirect, as mediated by enjoyment. These indirect effects are suggestive of the complex processes by which an alcohol-prevention serious game functions, with the concept of enjoyment at its core, which is consistent with media entertainment theory that posits that enjoyment is central to learning-related outcomes for entertainment media in general and serious games in particular [29].

These results on the mediating role of enjoyment suggest the need to integrate entertainment content, components, and appeals in serious game design and development as a means to foster learning and behavioral outcomes related to health. The combination of behavioral theory and game theory is needed to elicit desired health-related outcomes [19]. The design of our game is based on social cognitive theory [30], whereby game players learn through the social modeling of nonplayer characters, and PMT, which emphasizes the importance of threat and coping [21]. In addition, game theory specifies that a storytelling format is required to engage players in a serious game [9,10]. Important here is that game players have a degree of control in their decision making and subsequent trajectory across the game experience, which likely contributes to their level of game enjoyment.

It is also important to consider the role of AUDIT-C in the process. Participants with higher scores on AUDIT-C selected more in-game risky alcohol decisions, which is emblematic of how in-game decisions mirror real-world behaviors. In turn, participants who made more risky decisions enjoyed the game less. In terms of the overall process, participants with higher levels of AUDIT-C enjoyed the game less and, via the indirect pathway, had lower levels of intention to drink less and drinking refusal self-efficacy. That achieving positive health outcomes is impeded in this manner for our sample’s most hazardous drinkers (ie, those with high AUDIT-C levels) may relate to message discrepancy [51], which involves how the adoption of a recommended behavior (eg, decreased alcohol consumption) is most likely to occur when one’s own current behavior is not that dissimilar from the recommended behavior. Thus, because the drinking behaviors of participants with higher scores on AUDIT-C are in sharp contrast with the game’s recommended behavior, these participants are less likely to develop beneficial outcomes.

### Limitations

We consider 4 main limitations here. First, while we assessed game time and risky alcohol decisions, there may be other in-game factors at play, including presence, flow, and engagement, which should be considered in future research. Second, our conceptual model included only one individual characteristic, AUDIT-C. Future research may want to use the full AUDIT because it has greater specificity in identifying hazardous drinking. Another individual factor that should be considered in future research is impulsivity, which is a well-recognized risk factor for both the initiation and continuation of alcohol misuse [52]. Third, another limitation of our study is that, while the one-group, pretest-posttest, quasi-experimental design permitted us to implement auto-regression models, an experimental design with a control group would be more robust. Fourth, we only assessed the short-term effects of the video game, but research suggests that the behavioral outcomes of serious health games may diminish with time [19]. To address this concern, it is recommended that future research measure both short-term and long-term outcomes.

### Conclusions

Using the serious video game One Shot, we built and tested a multistep model for serious video game processes. The confirmation of research hypotheses suggests that serious video games can influence the alcohol consumption outcomes of young adults aged 21 to 25 years, which is an understudied at-risk group. Enjoyment is at the core of the documented processes, serving as an essential step in explaining how alcohol-related outcomes result from individuals’ differential hazardous drinking levels and in-game decisions. This integral role of enjoyment has theoretical implications for researchers of serious video games and practical implications for game designers and developers.

## Abbreviations

AUDIT-C: Alcohol Use Disorders Identification Test-Concise
CFA: confirmatory factor analysis
CFI: comparative fit index
PMT: protection motivation theory
RMSEA: root mean square error of approximation
RQ: research question
SEM: structural equation model
SSI: Survey Sampling International
